# Alkyl-quinolone-dependent quorum sensing controls prophage-mediated autolysis in *Pseudomonas aeruginosa* colony biofilms

**DOI:** 10.3389/fcimb.2023.1183681

**Published:** 2023-05-26

**Authors:** Giulia Giallonardi, Morgana Letizia, Marta Mellini, Emanuela Frangipani, Nigel Halliday, Stephan Heeb, Miguel Cámara, Paolo Visca, Francesco Imperi, Livia Leoni, Paul Williams, Giordano Rampioni

**Affiliations:** ^1^ Department of Science, University Roma Tre, Rome, Italy; ^2^ National Biofilms Innovation Centre, Biodiscovery Institute and School of Life Sciences, University of Nottingham, Nottingham, United Kingdom; ^3^ IRCCS Fondazione Santa Lucia, Rome, Italy; ^4^ NBFC, National Biodiversity Future Center, Palermo, Italy

**Keywords:** *Pseudomonas aeruginosa*, quorum sensing, autolysis, biofilm, prophage, PQS, PqsL, Pf4

## Abstract

*Pseudomonas aeruginosa* is a model quorum sensing (QS) pathogen with three interconnected QS circuits that control the production of virulence factors and antibiotic tolerant biofilms. The *pqs* QS system of *P. aeruginosa* is responsible for the biosynthesis of diverse 2-alkyl-4-quinolones (AQs), of which 2-heptyl-4-hydroxyquinoline (HHQ) and 2-heptyl-3-hydroxy-4(*1H*)-quinolone (PQS) function as QS signal molecules. Transcriptomic analyses revealed that HHQ and PQS influenced the expression of multiple genes via PqsR-dependent and -independent pathways whereas 2-heptyl-4-hydroxyquinoline *N*-oxide (HQNO) had no effect on *P. aeruginosa* transcriptome. HQNO is a cytochrome *bc*_1_ inhibitor that causes *P. aeruginosa* programmed cell death and autolysis. However, *P. aeruginosa pqsL* mutants unable to synthesize HQNO undergo autolysis when grown as colony biofilms. The mechanism by which such autolysis occurs is not understood. Through the generation and phenotypic characterization of multiple *P. aeruginosa* PAO1 mutants producing altered levels of AQs in different combinations, we demonstrate that mutation of *pqsL* results in the accumulation of HHQ which in turn leads to Pf4 prophage activation and consequently autolysis. Notably, the effect of HHQ on Pf4 activation is not mediated *via* its cognate receptor PqsR. These data indicate that the synthesis of HQNO in PAO1 limits HHQ-induced autolysis mediated by Pf4 in colony biofilms. A similar phenomenon is shown to occur in *P. aeruginosa* cystic fibrosis (CF) isolates, in which the autolytic phenotype can be abrogated by ectopic expression of *pqsL*.

## Introduction

The ubiquitous and metabolically versatile opportunistic pathogen *Pseudomonas aeruginosa* is a common cause of difficult-to-eradicate chronic lung infections in individuals with cystic fibrosis (CF), a genetically inherited disease characterized by a considerable increase in the viscosity of the airway secretions, which favours bacterial colonization (Lyczak et al., 2000). In the CF lung, *P. aeruginosa* is subjected to a challenging environment, mainly due to antibiotic therapy and to the interaction with co-infecting species, bacteriophages, and host immune defences including macrophages and neutrophils (Brockhurst et al., 2005; Jensen et al., 2010; McKeon et al., 2010; Filkins et al., 2015; James et al., 2015). The selective pressure over the bacterial population promotes diversification in the colonizing *P. aeruginosa* population, leading to the emergence of characteristic genetic variants. As a result, small colony variants, mucoid, rough, lipopolysaccharide-defective, or hyper-piliated morphotypes, as well as autolytic colony phenotypes, have often been observed in *P. aeruginosa* CF isolates (Dasgupta et al., 1994; Deretic et al., 1994; Martin et al., 1995; Haüssler et al., 1999; Déziel et al., 2001; D’Argenio et al., 2002).

As a form of regulated cell death, bacterial autolysis plays an important role in developmental processes, such as horizontal gene transfer, biofilm formation, and the elimination of damaged cells irreversibly injured by environmental or antibiotic stress (Allesen-Holm et al., 2006; Rice and Bayles, 2008). One of the best-characterized examples of regulated cell death in bacteria involves the transition of prophages to the lytic cycle. Autolysis has been frequently described as bacteriophage plaque-like zones of lysis in *P. aeruginosa* cultures on agar (Hadley, 1924; Berk, 1963; Berk, 1965; Holloway, 1969; Webb et al., 2003) and several studies have documented that high titers of filamentous Pf prophages often correlate with cell death in *P. aeruginosa* (Webb et al., 2003; Kirov et al., 2007; Rice et al., 2009). In addition, depending on the type and developmental stage, ‘explosive autolysis’ can occur in *P. aeruginosa* biofilms as a result of prophage endolysin-mediated peptidoglycan degradation (Turnbull et al., 2016; Hynen et al., 2021).

**Figure 1:**
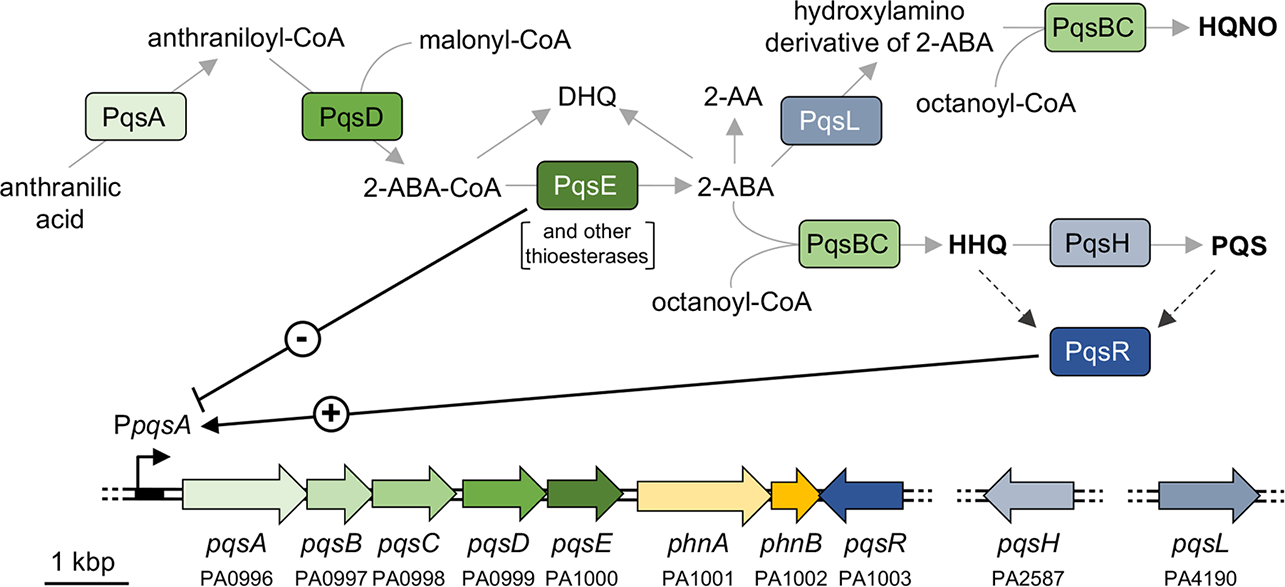
Schematic representation of the AQ biosynthetic pathway and the *pqs* and *phn* genes in *P. aeruginosa*. The major AQs (HHQ, PQS, and HQNO) are in bold face. The PA number is indicated below the genes according to the *Pseudomonas* Genome Database (Winsor et al., 2011). Solid grey arrows represent biosynthesis; dashed black arrows represent information flow; solid black arrow indicates activation (+); black T-line indicates negative regulation (-).

D’Argenio and co-workers reported that transposon insertions in the *pqsL* gene, coding for the PqsL monooxygenase, result in autolysis of *P. aeruginosa* PAO1 in colony biofilms and overproduction of the quorum sensing (QS) signal molecule 2-heptyl-3-hydroxy-4(*1H*)-quinolone (PQS), thus establishing a link between the lysis phenotype and the *pqs* QS system (D’Argenio et al., 2002). The *pqs* system depends on 2-alkyl-4-quinolone (AQ) signal molecules and is part of the QS regulatory network in *P. aeruginosa*, together with the two *N*-acylhomoserine lactone-based *las* and *rhl* QS systems (Lee and Zhang, 2015). The *pqs* system includes the transcriptional regulator PqsR (also known as MvfR), which positively regulates the expression of the *pqsABCDE-phnAB* operon upon binding to either 2-heptyl-4-hydroxyquinoline (HHQ) or PQS (**Figure 1**) (Ilangovan et al., 2013). The PqsA-D enzymes are involved in the synthesis of 4-hydroxyalkyl quinolines, called Series A congeners, that differ in length of the 2-position alkyl chain (Déziel et al., 2004). The most studied Series A congener is HHQ that is converted to PQS, belonging to Series B congeners, by the PqsH monooxygenase, encoded by the *pqsH* gene. A second monooxygenase, PqsL, is required together with the *pqsABCD* gene products for the synthesis of 2-heptyl-4-hydroxyquinoline *N*-oxide (HQNO) (Lépine et al., 2004). HQNO is a well-known cytochrome inhibitor that binds to the quinone reduction (Qi) site of the cytochrome *bc*_1_ complex disrupting the flow of electrons and resulting in the generation of reactive oxygen species. These can cause *P. aeruginosa* cell death and autolysis favouring biofilm formation and antibiotic tolerance (Hazan et al., 2016). *P. aeruginosa pqsL* mutants unable to produce HQNO were reported not to undergo autolysis (Hazan et al., 2016). How this relates to the colony biofilm autolysis phenotype observed for *pqsL* mutants by D’Argenio and colleagues (D’Argenio et al., 2002) is not understood. However wild type strains naturally lyse in planktonic cultures and in biofilms implying the existence of HQNO-dependent and HQNO-independent autolysis pathways. Indeed, explosive autolysis does not depend on AQ production (Turnbull et al., 2016).

To discriminate between the regulons controlled by the AQs HHQ, PQS and HQNO in *P. aeruginosa*, we undertook a transcriptomic analysis in a *P. aeruginosa* PAO1 mutant strain impaired in AQ synthesis and conversion, grown in the presence of synthetic HHQ, PQS or HQNO (Rampioni et al., 2016). While HHQ and PQS were shown to directly or indirectly control the expression of hundreds of genes, HQNO did not affect the *P. aeruginosa* transcriptome, indicating that HQNO is not a signal molecule (Rampioni et al., 2016). The potentially contradictory observations surrounding HQNO and the autolytic phenotype of *pqsL* mutants (D’Argenio et al., 2002; Hazan et al., 2016), prompted us to investigate the link between the *pqs* QS system and autolysis. Here we provide evidence that *pqsL* mutation causes autolysis of *P. aeruginosa* PAO1 in colony biofilms as a consequence of HHQ accumulation, that in turn leads to the conversion of the Pf4 prophage to a lytic form. Interestingly, *pqsL* mutations also appear to be the primary cause of autolysis in colony biofilms of *P. aeruginosa* CF isolates.

## Materials and methods

### Bacterial strains, culture conditions and chemicals

Laboratory bacterial strains and *P. aeruginosa* CF isolates used in this study are listed in **Tables S1**, **S2**, respectively. The CF isolates belong to three different collections of bacterial strains isolated from respiratory secretions (*i.e.*, sputum, hypopharyngeal aspirate, bronchoalveolar lavage) of CF patients. Thirty-two clinical isolates were provided from CF patients in follow-up at the Cystic Fibrosis Centre of the Bambino Gesù Children’s Hospital in Rome, Italy (Imperi et al., 2019; Baldelli et al., 2020; Collalto et al., 2022), 12 isolates were from the Cystic Fibrosis Clinic in Hannover, Germany (Bragonzi et al., 2009), and 6 clinical isolates were from CF patients at the Cystic Fibrosis Centre of the Sapienza University in Rome, Italy (Massai et al., 2011). *Escherichia coli* and *P. aeruginosa* strains were cultured in Lysogeny Broth medium (LB), either in broth or on plates supplemented with 1.5% (w/v) agar (Sambrook et al., 1989). Unless otherwise stated, antibiotics were added at the following concentrations: *E. coli*, 30 μg mL^-1^ chloramphenicol (Cm), 10 μg mL^-1^ tetracycline (Tc), 10 μg mL^-1^ gentamicin (Gm); *P. aeruginosa*, 375 μg mL^-1^ Cm, 100 μg mL^-1^ Tc, 100 μg mL^-1^ Gm. When required the medium was supplemented with 1 mM isopropyl-β-D-1-thiogalactopyranoside (IPTG). All AQs were synthesized in-house as described by Ortori and collaborators (Ortori et al., 2011). Stock solutions of AQs (10 mM) were prepared in MeOH.

### Recombinant DNA techniques

The plasmids and oligonucleotides used in this study are listed in **Tables S3**, **S4**, respectively. Preparation of plasmid DNA, purification of DNA fragments, restriction enzyme digestions, ligations, and transformations in *E. coli* DH5α or S17.1λ*pir* competent cells were performed with standard procedures (Sambrook et al., 1989). DNA amplification was performed by Polymerase Chain Reaction (PCR) using the GoTaq Polymerase (Promega). FastDigest restriction enzymes were purchased from Thermo Fisher Scientific. The ligation of DNA fragments was performed with the T4 DNA ligase (Promega). Plasmids were introduced into *P. aeruginosa* by transformation or by bi-parental conjugation using *E. coli* S17.1λ*pir* as the donor strain (Sambrook et al., 1989). All the plasmids generated in this study were verified by restriction analysis and DNA sequencing, and details on their construction are given in **Table S3**.

### Generation of *P. aeruginosa* mutants

*P. aeruginosa* deletion mutants were constructed by allelic exchange using pDM4-derivative plasmids, as previously described (Milton et al., 1996). The construction of pDM4-derivative plasmids is described in **Table S3**. Plasmids were independently introduced into *P. aeruginosa* strains following conjugal mating with *E. coli* S17.1λ*pir* as the donor strain (Sambrook et al., 1989). Clones with a chromosomal insertion of the pDM4-derivative plasmids were selected on LB agar plates supplemented with 375 µg mL^-1^ Cm and 15 µg mL^-1^ nalidixic acid. Plasmid excision from the chromosome was subsequently selected on LB agar plates supplemented with 10% (w/v) sucrose. The resulting mutations were confirmed by PCR analysis.

### Lysis phenotype and phage quantification

The lysis phenotype was evaluated using the colony biofilm assay and soft-agar lawns. For colony biofilm assays, 5 μL of overnight cultures grown at 37°C in LB were spotted onto Congo Red plates [10 g L^-1^ Tryptone (Acumedia), 15 g L^-1^ Bacto Agar (Difco), 40 mg L^-1^ Congo Red (VWR International), 10 mg L^-1^ Coomassie Brilliant Blue R-250 (Fluka)] and incubated for 24 h at 37°C. When required, 1 mM IPTG was added to the plates.

For soft-agar lawns, 100 μL of *P. aeruginosa* overnight cultures grown at 37°C in LB were diluted in 5 mL of LB Top Agar (LB-TA) [LB supplemented with 7.5 g L^-1^ Bacto Agar (Difco)]. Cultures in LB-TA were spread onto LB agar plates. When required, 5 μL of 10 mM C_5_ (*i.e.*, 2-pentyl-4-hydroxyquinoline; PHQ), C_7_ (*i.e.*, HHQ), C_9_ (*i.e.*, 2-nonyl-4-hydroxyquinoline; NHQ), or C_11_ (*i.e.*, 2-undecyl-4-hydroxyquinoline; UHQ) Series A congeners, 2,4-dihydroxyquinoline (DHQ), PQS, or methanol (as control) were spotted on the soft-agar lawns before incubating for 24 h at 37°C.

Phage quantification was performed as previously described (Frangipani, 2014). Briefly, soft-agar lawns, prepared as described above, were scraped from the plate and suspended in 2 mL of TNM buffer [1.21 g L^-1^ Tris, 8.77 g L^-1^ NaCl, 2.46 g L^-1^ MgSO_4_ · 7H_2_O; pH 7.4]. After vortexing, the filtered supernatant was recovered and sequentially diluted in TNM buffer. Five-μL of each dilution were spotted onto soft-agar inoculated with the *P. aeruginosa* ΔPf4-K mutant (**Table S1**) (Rice et al., 2009), and Plaque Forming Units (PFU) were counted. The average data and standard deviations were calculated from at least three independent experiments.

### Quantification of AQ production

AQs produced by *P. aeruginosa* PAO1, the corresponding isogenic mutants, and CF lytic strains carrying the pME6032 or pME-*pqsL* plasmids were quantified by LC-MS/MS analysis, as previously described (Ortori et al., 2011). Briefly, 1 mL of filtered supernatant from overnight cultures grown at 37°C in LB or in LB supplemented with 1 mM IPTG was extracted with ethyl acetate, dried under vacuum, and redissolved in methanol prior to LC-MS/MS. AQs in cell-free supernatants prepared from *P. aeruginosa* PAO1 and diverse clinical isolates were also determined using the reporter strain AQ-Rep, as previously described (Fletcher et al., 2007; Mellini et al., 2019). Briefly, *P. aeruginosa* cultures were grown overnight in LB at 37°C with shaking (200 rpm). Following overnight growth, bacteria were diluted to an optical density at 600 nm (OD_600_) of 0.01 in 5 mL of LB or LB supplemented with 1 mM IPTG and grown at 37°C with shaking. Supernatants were collected after 16 h of growth. Quantification of AQs was performed by adding 5 µL of cell-free culture supernatant to 195 µL of cultures of the AQ-Rep strain (OD_600_ = 0.1) in 96-well, black, clear-bottomed microtiter plates. The microtiter plates were incubated at 37°C. Light emission (relative light units, RLU) and cell density (OD_600_) were measured after 6 h of incubation using an automated luminometer-spectrophotometer plate reader Spark 10M (Tecan). AQ-Rep activity was determined as the RLU value normalized to the corresponding OD_600_. A calibration curve was generated by growing the reporter strain in the presence of increasing concentrations of PQS. The resulting dose-response curve was used to extrapolate the concentration of AQs in the culture supernatants.

For both LC-MS/MS analysis and the assays performed by using the AQ-Rep reporter strain, the average data and standard deviations were calculated from at least three independent experiments.

### Promoter activity assays

Overnight cultures of *P. aeruginosa* PAO1 strains carrying chromosomal P*pqsA::lux* fusion (Diggle et al., 2007) were diluted to an OD_600_ of 0.01 in LB, and 200 µL cultures were grown at 37°C in 96-well, black, clear-bottomed microtiter plates. RLU and OD_600_ were measured using an automated luminometer-spectrophotometer plate reader Spark 10M (Tecan) every h to determine maximal promoter activity. Promoter activity is given as RLU divided by OD_600_. The average data and standard deviations were calculated from at least three independent experiments.

### RNA extraction and reverse transcription-quantitative PCR

RNA was extracted as previously described (Rampioni et al., 2016) from colony biofilms of *P. aeruginosa* PAO1, Δ*pqsL*, Δ*pqsHL*, Δ*pqsAL* and Δ*pqsAHL* grown at 37°C on Congo Red plates.

For each sample, three different pools of RNA were extracted in independent experiments (biological triplicates). Briefly, cells resuspended in sterile saline [0.9% (w/v) NaCl] to an OD_600_ of 1.0 were mixed with 2 mL RNA Protect Bacteria Reagent (Qiagen), and RNA was purified using the RNeasy mini-kit (Qiagen) including the on-column DNase I digestion step. In addition, eluted RNA was treated for 1 h at 37°C with TURBO DNase (0.2 U per µg of RNA; Ambion) and with SUPERase-In (0.4 U per µg of RNA; Ambion). DNase I was removed with the RNeasy Column purification kit (Qiagen). Purified RNA was quantified using the NanoDrop 2000 spectrophotometer (Thermo Fisher Scientific). The absence of contaminating chromosomal DNA was verified by PCR using the oligonucleotides FW*pqsC*UP and RV*pqsC*UP (**Table S4**).

For RT-qPCR analyses, cDNA synthesis was performed from 1 µg of purified RNA using the iScript Reverse Transcription Supermix for RT-qPCR kit (Bio-Rad). Real-time PCRs were performed using the iTaq Universal SYBR Green Supermix (Bio-Rad) and the Rotor Gene 6000 Thermocycler (Corbett Research). Gene-specific primers employed in this analysis (**Table S4**) were designed using Primer-BLAST software (www.ncbi.nlm.nih.gov/tools/primer-blast) to avoid nonspecific amplification of *P. aeruginosa* DNA. 16S rRNA was chosen as the internal control to normalize the real-time PCR data in every single run (Letizia et al., 2022) and to calculate the relative FC in gene expression using the 2^–ΔΔCt^ method. Average values and standard deviations were calculated from three biological replicates.

### Statistical analysis

Statistical analysis was performed with GraphPad Prism 6.01 software using one-way analysis of variance followed by Tukey-Kramer multiple-comparison test (ANOVA) or the unpaired t-test depending on the experiment. Differences with a *P* value of < 0.05 were considered statistically significant.

## Results

### Colony biofilm of the Δ*pqsL* mutant undergoes autolysis as a consequence of HHQ accumulation

**Figure 2:**
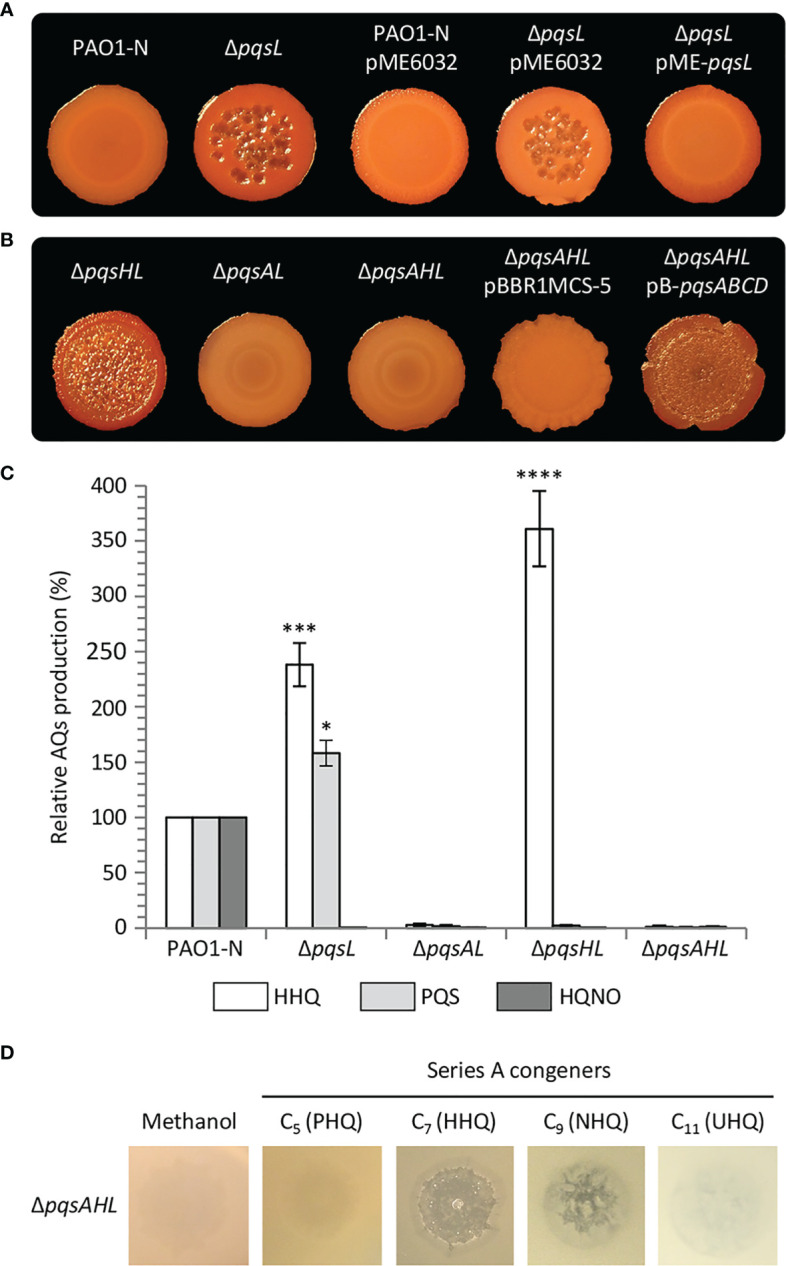
**(A, B)** Colony biofilms on Congo-Red agar plates formed by wild type *P. aeruginosa* PAO1-N or the indicated isogenic mutants. The medium was supplemented with 1 mM IPTG for the strains carrying the pME6032 or pME-*pqsL* plasmids. **(C)** Histogram reporting the levels of HHQ, PQS and HQNO produced by the indicated strains grown in LB. The average of three independent experiments is reported with standard deviation (SD). *P < 0.05; ***P < 0.001; ****P < 0.0001. **(D)** Images of soft-agar lawns formed by the *P. aeruginosa* PAO1-N Δ*pqsAHL* mutant treated with 5 µL of 10 mM C_5_ (PHQ), C_7_ (HHQ), C_9_ (HNQ) or C_11_ (UHQ) Series A congeners, or methanol as the control. For **(A, B, D)**, representative pictures of three independent experiments are shown.

To investigate the role played by PqsL in *P. aeruginosa* autolysis, we generated a *pqsL* in-frame deletion mutant in *P. aeruginosa* PAO1-N (PAO1 from the Nottingham collection). In common with the *pqsL* phenotype observed for *P. aeruginosa* PAO1 (Iglewski collection) (D’Argenio et al., 2002) mutation of *pqsL* in PAO1-N caused autolysis in both colony biofilms (**Figure 2A**) and in soft-agar lawn (**Figure S1**). This contrasts with the smooth phenotype with no visible lysis shown by the parental strain grown under the same conditions. The autolysis phenotype could be genetically complemented by ectopic expression of *pqsL via* the pME6032-derived plasmid pME-*pqsL* (**Figures 2A**; **S1**). Autolysis likely occurs only on solid media, as the growth kinetics of wild type PAO1-N and its isogenic Δ*pqsL* mutant were comparable in liquid cultures (**Figure S2**).

It was suggested that mutation of *pqsL* provokes autolysis as a consequence of PQS accumulation (D’Argenio et al., 2002). However, in our hands, the lytic phenotype was even more evident in a *P. aeruginosa* Δ*pqsHL* double mutant, unable to synthesize PQS as a consequence of *pqsH* deletion, than in the Δ*pqsL* mutant (**Figures 2B**; **S1**). Conversely, no autolysis was detected when the *pqsA* gene was deleted in the lytic Δ*pqsL* and Δ*pqsHL* genetic backgrounds (*i.e*., in the Δ*pqsAL* and Δ*pqsAHL* mutant strains; **Figures 2B**; **S1**). These data argue against the hypothesis that autolysis is induced by PQS and suggest that this phenotype depends on an AQ whose synthesis requires PqsA, but not PqsH or PqsL.

Since the synthesis of the AQs HHQ, PQS and HQNO requires the same substrates (*i.e*., anthranilate and fatty acids; **Figure 1**) (Dulcey et al., 2013), it is likely that mutation of *pqsL* results in both HHQ and PQS overproduction. To clarify this issue, we quantified the HHQ, PQS and HQNO levels produced by the lytic strains Δ*pqsL* and Δ*pqsHL* and the non-lytic strains Δ*pqsAL* and Δ*pqsAHL*. HHQ levels in the tested strains paralleled the lysis phenotype, with the Δ*pqsL* and Δ*pqsHL* mutants producing about 2.5- and 3.6-fold higher levels of HHQ with respect to wild type PAO1-N (**Figure 2C**). These data highlight a correlation between HHQ accumulation and autolysis in strains unable to synthesize HQNO. A link between HHQ and autolysis was also corroborated by the evidence that this phenotype can be restored by the plasmid-driven expression of the genes required for HHQ synthesis *via* the pB-*pqsABCD* plasmid in the *P. aeruginosa* triple mutant Δ*pqsAHL*, impaired in AQ synthesis and conversion (**Figure 2B**).

The enzymes encoded by the *pqsABCD* genes are responsible for the synthesis of intermediates required for AQ production (**Figure 1**). PqsA converts anthranilate to anthraniloyl-coenzyme A (anthraniloyl-CoA) that is condensed with malonyl-CoA to form 2-aminobenzoylacetyl-CoA (2-ABA-CoA) in a reaction catalysed by PqsD (Coleman et al., 2008; Zhang et al., 2008). PqsE and other thioesterases convert 2-ABA-CoA into 2-aminobenzoylacetate (2-ABA) (Drees and Fetzner, 2015). This latter molecule can be condensed with octanoyl-CoA by the PqsBC heterodimer to form HHQ, or can be decarboxylated to form the volatile 2-aminoacetophenone (2-AA) (Dulcey et al., 2013; Drees et al., 2016). Moreover, 2-ABA can be spontaneously converted into DHQ (Zhang et al., 2008; Dulcey et al., 2013). Therefore, there was a possibility that the lysis phenotype observed in the Δ*pqsL* and Δ*pqsHL* mutants, as well as in the Δ*pqsAHL* strain carrying the pB-*pqsABCD* plasmid, may be due to the accumulation of 2-AA or DHQ. Consequently, the *pqsB* and *pqsC* genes were independently deleted in the Δ*pqsL* genetic background, generating the double mutants Δ*pqsLB* and Δ*pqsLC*. These latter strains are in principle capable of synthesizing 2-ABA, and hence 2-AA and DHQ, but unable to convert 2-ABA into HHQ. Independent deletion of either *pqsB* or *pqsC* in the Δ*pqsL* genetic background suppressed the lysis phenotype (**Figure S3**), confirming that autolysis depends on HHQ, or possibly by other Series A congeners produced *via* the *pqsABCD* biosynthetic pathway, while accumulation of HHQ precursors is not sufficient to result in autolysis.

Since the most abundant Series A congeners contain an odd carbon number alkyl chain (Déziel et al., 2004), we tested the ability of C_5_ (PHQ), C_7_ (HHQ), C_9_ (NHQ), and C_11_ (UHQ) Series A congeners to induce lysis when spotted onto soft-agar lawns of the *P. aeruginosa* Δ*pqsAHL* mutant, unable to synthesize AQs or to convert exogenously provided series A congeners into series B congeners. The PAO1 Δ*pqsAHL* strain showed visible lysis in the presence of HHQ and NHQ, but not when treated with PHQ and UHQ, or with methanol as control (**Figure 2D**). Since HHQ and NHQ are produced by wild type *P. aeruginosa* at similar concentrations (Lépine et al., 2004; Ortori et al., 2011), it is likely that the lytic strains Δ*pqsL* and Δ*pqsHL* accumulate both NHQ and HHQ, and hence that these Series A congeners both contribute to autolysis in the strains impaired in HQNO synthesis. However, for simplicity and considering that most QS studies focus on HHQ as the primary *P. aeruginosa* Series A congener and precursor of PQS, only HHQ was considered in further analyses.

### HHQ accumulation induces autolysis *via* PqsR- and PqsE-independent pathways

The primary role of HHQ when produced at wild type levels in *P. aeruginosa* is to drive the expression of the *pqsABCDE-phnAB* operon *via* PqsR, ultimately autoinducing in its own synthesis and the production of the PqsE effector protein (Rampioni et al., 2016). Notably, NHQ, in common with HHQ is also highly active in binding to and activating PqsR (Fletcher et al., 2007; Ilangovan et al., 2013).

**Figure 3:**
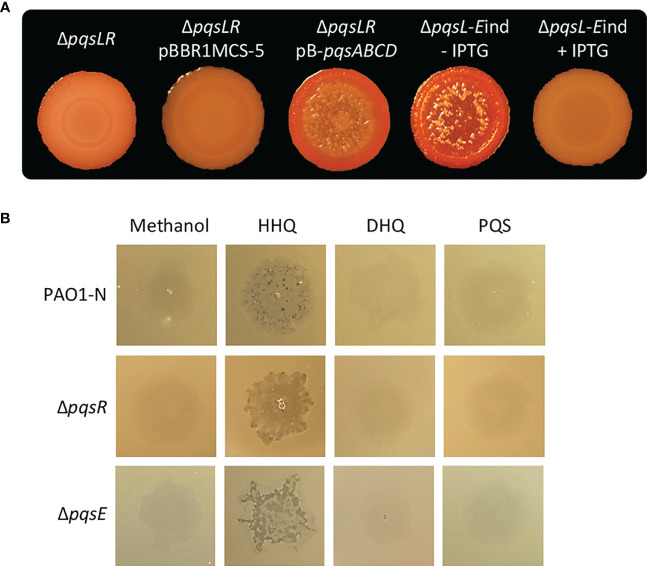
**(A)** Colony biofilms on Congo-Red agar plates formed by the indicated *P. aeruginosa* PAO1-N isogenic mutants. For the Δ*pqsL*-*E*ind strain, the medium was supplemented (+ IPTG) or not (- IPTG) with 1 mM IPTG. **(B)** Images of soft-agar lawns formed by wild type *P. aeruginosa* PAO1-N or the indicated isogenic mutants treated with 5 µL of 10 mM HHQ, DHQ, PQS, or methanol as a control. For **(A, B)**, representative images of three.

To investigate the possibility that the lysis phenotype caused by HHQ overproduction in the Δ*pqsL* mutant could be mediated by PqsR and/or PqsE, we first deleted the *pqsR* gene in the lytic strain *P. aeruginosa* Δ*pqsL*. As shown in **Figure 3A**, the resulting Δ*pqsLR* mutant did not undergo autolysis in plate assays. However, PqsR is required for the transcriptional activation of the *pqsABCDE-phnAB* operon, and hence for the production of both HHQ and PqsE (Gallagher et al., 2002; Déziel et al., 2004), irrespective of the presence or the absence of the *pqsL* gene. In fact, P*pqsA* promoter activity was abrogated in the Δ*pqsLR* mutant strain (**Figure S4**). Notably, the autolysis phenotype could be restored in the Δ*pqsLR* double mutant by *in trans* constitutive expression of the *pqsABCD* genes, required for HHQ synthesis (**Figure 3A**), demonstrating that PqsR and, likely, PqsE are dispensable for the HHQ-induced autolysis. The evidence that HHQ accumulation provokes autolysis independently of PqsR and PqsE is corroborated by experiments performed on soft-agar lawns, showing that exogenous provision of HHQ promotes lysis also in the Δ*pqsR* and Δ*pqsE* mutants (**Figure 3B**).

Considering the negative effect exerted by PqsE on *pqsABCDE-phnAB* transcription (Hazan et al., 2010; Rampioni et al., 2010), we hypothesized that PqsE could alleviate HHQ-induced autolysis. To clarify this issue, the *pqsE* promoter was replaced with an IPTG-inducible one (Rampioni et al., 2010) in the chromosome of the *P. aeruginosa* lytic strain Δ*pqsL*, resulting in the generation of the Δ*pqsL-E*ind conditional mutant. Interestingly, the lack of *pqsE* expression in the lytic strain resulted in more pronounced autolysis with respect to the PqsE-proficient background (compare the Δ*pqsL* mutant in **Figure 2A** with the Δ*pqsL*-*E*ind mutant in **Figure 3A**). This is in line with the increased activity of the P*pqsA* promoter in the Δ*pqsL*-*E*ind mutant grown in the absence of IPTG with respect to the Δ*pqsL* background (**Figure S4**). On the contrary, the IPTG-induced overexpression of *pqsE* completely abrogated autolysis and P*pqsA* activity in the Δ*pqsL*-*E*ind mutant (**Figures 3A**; **S4**).

Overall, these data demonstrate that HHQ overproduction in the Δ*pqsL* strain causes autolysis *via* a PqsR- and PqsE-independent mechanism(s), and suggest a potential homeostatic role for PqsE in alleviating autolysis in the mutant strains unable to synthesize HQNO.

### Autolysis in the Δ*pqsL* mutant is mediated by the Pf4 prophage

**Figure 4:**
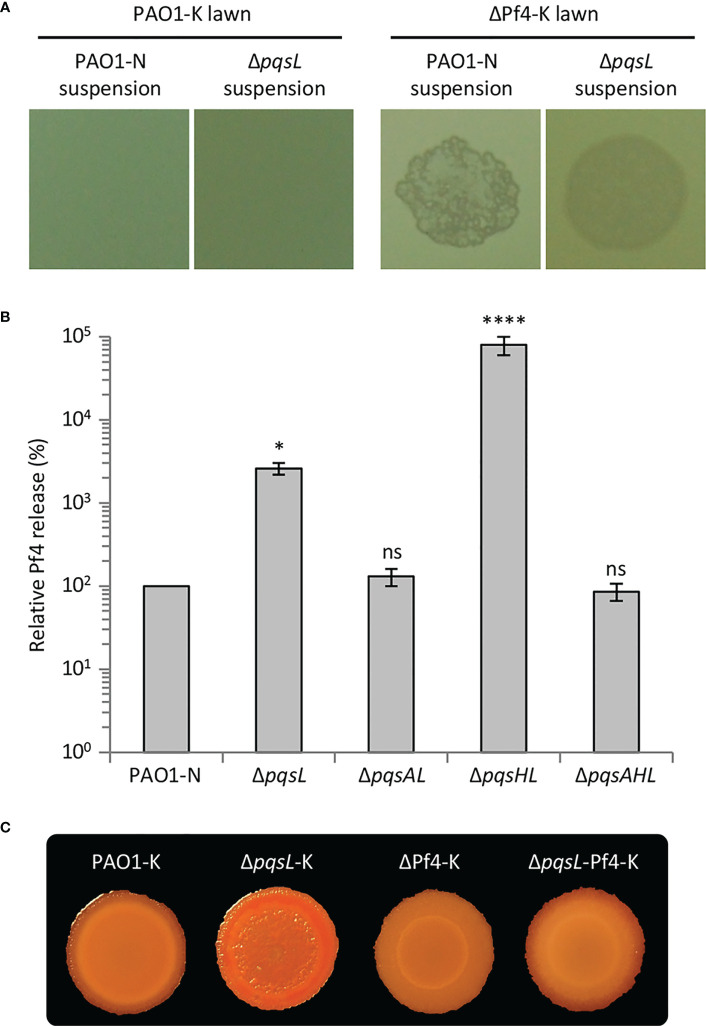
**(A)** Images of soft-agar lawns formed by wild type *P. aeruginosa* PAO1-K or its isogenic mutant deleted in the Pf4 prophage (ΔPf4-K) treated with 5 µL of filtered supernatants from overnight LB cultures of wild type *P. aeruginosa* PAO1-N or it isogenic Δ*pqsL* mutant. **(B)** Histogram reporting the relative Pf4 release in soft-agar lawns formed by wild type *P. aeruginosa* PAO1-N or the indicated isogenic mutants. PFU count from the soft-agar lawn of PAO1 is arbitrarily taken as 100%. The average of five independent experiments is reported with standard deviation (SD). *P < 0.05; ****P < 0.0001; ns, not statistically significant. **(C)** Colony biofilms on Congo-Red agar plates formed by the indicated strains. For **(A, C)**, representative images of three independent experiments are shown.

The autolysis observed in the *P. aeruginosa* Δ*pqsL* mutant strain resembles plaques formed as a consequence of lytic phage release. *P. aeruginosa* PAO1 has two prophages integrated in its genome, Bacto1 and Pf4 (Stover et al., 2000; Finnan et al., 2004). Since Pf4 plays a pivotal role in *P. aeruginosa* autolysis during biofilm development (Webb et al., 2003; Kirov et al., 2007; Rice et al., 2009), we investigated the levels of Pf4 particles released by the lytic and non-lytic mutants. To this end, we made use of a *P. aeruginosa* PAO1 wild type strain and an isogenic Pf4 mutant where the entire prophage has been deleted (herein after referred to as PAO1-K and ΔPf4-K, respectively). PAO1-K is resistant to re-infection by Pf4, while Pf4 infection provokes lysis in the ΔPf4-K mutant; therefore, the presence of Pf4 in *P. aeruginosa* culture supernatants or biofilms can be in principle assayed by testing their ability to induce plaque formation when spotted onto soft-agar lawns of *P. aeruginosa* ΔPf4-K, but not of *P. aeruginosa* PAO1-K (Rice et al., 2009). Indeed, suspensions derived from soft-agar lawns formed by the *P. aeruginosa* PAO1-N wild type and Δ*pqsL* mutant provoked lysis in the ΔPf4-K mutant but not in the parental strain, indicating Pf4 release from the tested strains (**Figure 4A**).

The Pf4-dependent lysis assay was used to compare the number of Pf4 particles released by soft-agar lawns formed by the lytic and non-lytic *P. aeruginosa* strains. As shown in **Figure 4B**, Pf4 was released at the same level from the wild type, Δ*pqsAL* and Δ*pqsAHL* strains, indicating that HHQ levels produced by wild type PAO1-N do not affect Pf4 release. Conversely, the lytic strains Δ*pqsL* and Δ*pqsHL* released about 30- and 800-fold higher levels of Pf4 compared to the non-lytic strains, respectively (**Figure 4B**). The high level of Pf4 release in soft-agar lawns of the lytic strains clearly parallels their increased HHQ levels (**Figure 2C**), strongly suggesting that the autolysis phenotype observed in the HHQ-overproducing strains is caused by a transition of the Pf4 prophage to a lytic form.

In line with the evidence that HHQ-overproduction does not result in growth defects of the lytic strains in liquid planktonic cultures (**Figure S2**), the release of Pf4 particles was comparable in lytic (*i.e.*, Δ*pqsL* and Δ*pqsHL*) and non-lytic (*i.e.*, wild type, Δ*pqsAL* and Δ*pqsAHL*) strains when grown in LB broth with shaking.

To confirm the role played by Pf4 in autolysis, we introduced the Δ*pqsL* mutation into the *P. aeruginosa* PAO1-K and ΔPf4-K strains, thus generating the Δ*pqsL*-K and Δ*pqsL*-Pf4-K mutants, respectively. Deletion of *pqsL* in PAO1-K caused colony biofilm autolysis also in this genetic background (**Figure 4C**), as in PAO1-N (**Figure 2A**). Conversely, colony biofilms did not undergo autolysis when the *pqsL* deletion was introduced in the ΔPf4-K mutant (**Figure 4C**), unequivocally demonstrating that the Pf4 prophage is responsible for autolysis as a consequence of HHQ accumulation in Δ*pqsL* mutants.

### PqsL mutation is responsible for colony biofilm autolysis in *P. aeruginosa* CF isolates

**Figure 5:**
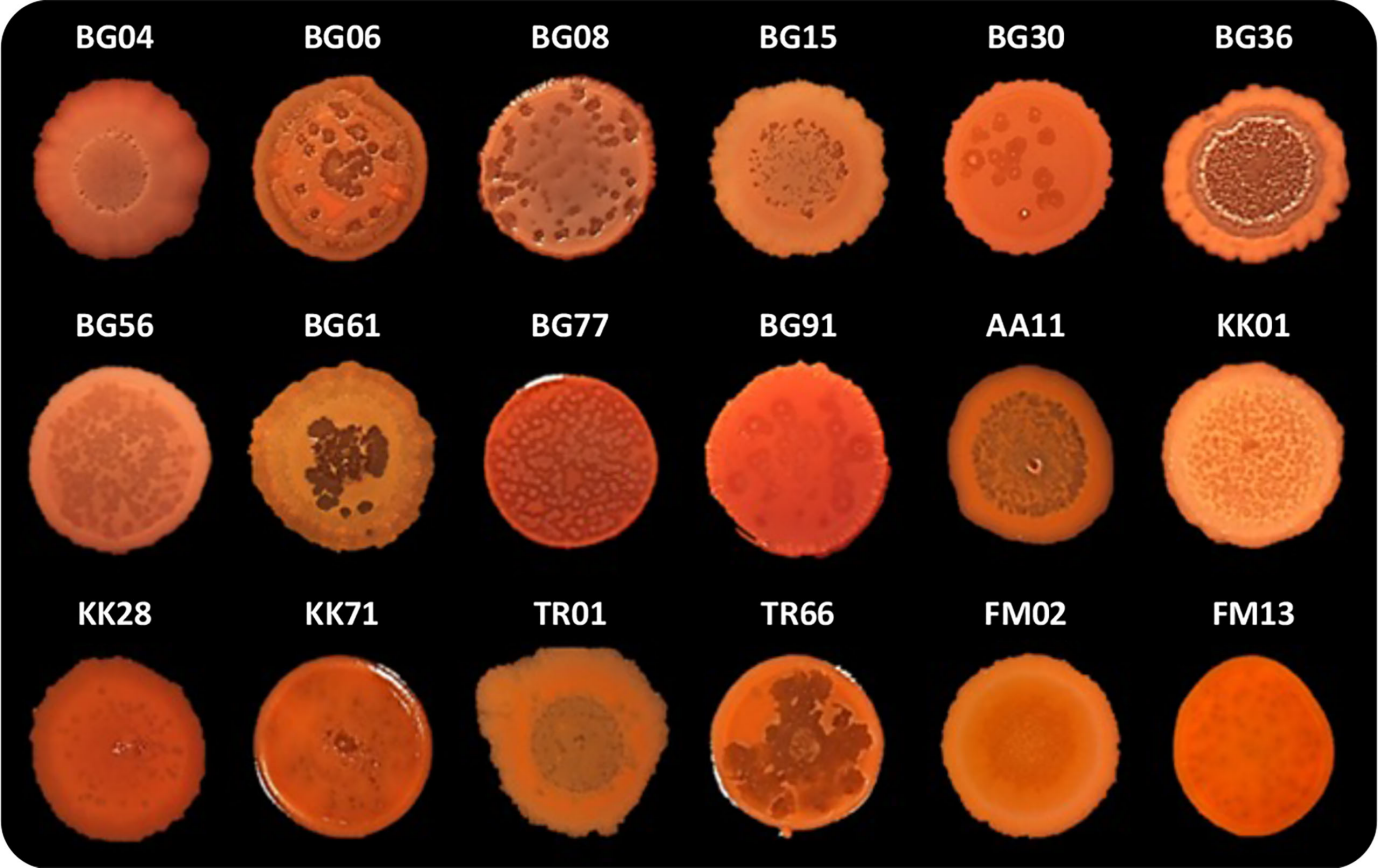
Colony biofilms on Congo-Red agar plates formed by the indicated *P. aeruginosa* CF isolates. Representative images of three independent experiments are shown.

As previously mentioned, *P. aeruginosa* CF isolates often display a lysis phenotype similar to PAO1 Δ*pqsL* when grown on solid media (D’Argenio et al., 2002; O’Connor et al., 2022). We thus decided to investigate autolysis in a collection of 50 P*. aeruginosa* strains isolated from the sputum of CF patients (**Table S2**). Eighteen out of 50 CF isolates (36%) displayed an autolysis phenotype when grown as colony biofilms. Autolysis was more evident in some clinical isolates, in which phage-like plaques were scattered over the whole surface of the colony biofilm (*i.e.*, in the BG06, BG08, BG56, BG77, KK01, KK71, and TR66 isolates) or were mainly located in the central area of the colony biofilm (*i.e.*, in the BG04, BG15, BG36, BG61, AA1, TR01, and FM02 isolates), than in others (*i.e.*, in the BG30, BG91, KK28, and FM13 isolates) (**Figure 5**).

In order to investigate the possible involvement of *pqsL* mutation in the lysis phenotype, the *pqsL* gene was PCR amplified from the genomes of the 18 lytic strains. Amplicons with expected molecular weight were obtained in 14 out of 18 lytic strains (77.8%), as well as in wild type PAO1-N used as control, while no amplicons were obtained in the CF isolates BG04, BG08, BG61, and FM13 despite the use of different primer pairs designed to amplify the whole *pqsL* gene or internal regions (**Figure S5**). These data suggest that in these 4 isolates the *pqsL* sequence may be absent or rearranged. The *pqsL* amplicons obtained from the remaining 14 CF isolates showing the lysis phenotype were sequenced and compared with the *pqsL* sequence of PAO1-N, which displays 100% identity to the *P. aeruginosa* PAO1 *pqsL* sequence retrieved in the *Pseudomonas* Genome Database (Winsor et al., 2011). The 18 CF lytic strains encompass isolates in which the *pqsL* gene could not be amplified (BG04, BG08, BG61, and FM13), isolates that encode a PqsL protein identical to that of PAO1-N (BG36, BG56, BG77, and FM02), and isolates that encode PqsL variants with single or multiple amino acid substitutions with respect to the PAO1-N protein (BG06, BG15, KK01, KK28, KK71, TR01, TR66, BG91, BG30, and AA11) (**Table S2**). Obviously, the identified amino acid substitutions are not predictive of PqsL reduced/abolished functionality. On the other hand, the presence of a functional *pqsL* allele does not guarantee production of the PqsL enzyme at physiological levels.

**Figure 6:**
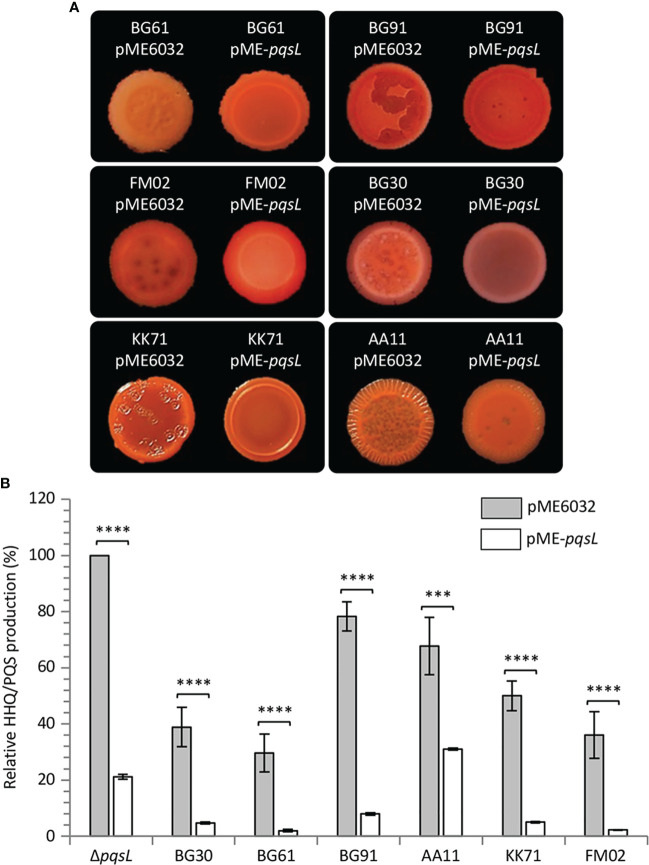
**(A)** Colony biofilms on Congo-Red agar plates supplemented with 1 mM IPTG formed by the indicated *P. aeruginosa* CF isolates carrying the pME6032 or pME-*pqsL* plasmids. Representative pictures of three independent experiments are shown. **(B)** Histogram showing the relative production of the AQs HHQ and PQS in filtered supernatants collected from the indicated strains carrying the pME6032 (grey bars) or the pME-*pqsL* (white bars) plasmids, grown in LB supplemented with 1 mM IPTG. The HHQ/PQS level produced by the *P. aeruginosa* Δ*pqsL* mutant carrying the pME6032 plasmid is arbitrarily taken as 100%. The average of three independent experiments is reported with standard deviation (SD). ***P < 0.001; ****P < 0.0001.

To provide genetic evidence of the correlation between altered PqsL functionality or *pqsL* expression and autolysis, the pME-*pqsL* plasmid for IPTG-dependent expression of wild type *pqsL* and the pME6032 empty vector were independently introduced in six representative isolates, including a strain that apparently lacks the *pqsL* gene (BG61), a strain with a functional *pqsL* allele (FM02), and strains that encode different PqsL variants (KK71, BG91, BG30, and AA11) (**Table S2**). Notably, colony biofilm autolysis was abrogated or severely attenuated in all the lytic strains carrying the pME-*pqsL* plasmid relative to the same strains carrying the pME6032 empty vector (**Figure 6A**). Moreover, in line with the results obtained for PAO1-N, a significant reduction in HHQ/PQS levels was observed in the CF strains carrying pME-*pqsL* relative to the same strains carrying pME6032 (**Figure 6B**). For what concerns HHQ production, this trend was confirmed by means of LC-MS/MS analysis (**Figure S6**). Overall, it is reasonable to propose that reduced *pqsL* expression and/or PqsL functionality are likely to be common drivers of colony biofilm autolysis in *P. aeruginosa* CF isolates.

## Discussion

Autolysis is one of the phenotypic adaptations that characterize *P. aeruginosa* CF strains, a property noted since early observations of clinical isolates and described as formation of colonies that lysed at their centre, characterized by plaque-like clearing developed in the areas of highest cell density in lawns (Hadley, 1924). Thenceforth, the autolysis phenotype has been described in different studies focused on *P. aeruginosa* clinical isolates (Berk, 1963; Berk, 1965; Holloway, 1969; D’Argenio et al., 2002; Webb et al., 2003; O’Connor et al., 2022). For example, D’Argenio and collaborators reported 31% (59 out of 191) of lytic strains in a collection of *P. aeruginosa* CF isolates (D’Argenio et al., 2002). In agreement with these observations, we found in the present study that in a set of 50 CF isolates, 36% displayed visible lysis when grown as colony biofilms. This lysis phenotype has long been observed in *P. aeruginosa* laboratory strains, especially in *pqs* mutants (D’Argenio et al., 2002; Déziel et al., 2004; Hazan et al., 2016; O’Connor et al., 2022). D’Argenio and collaborators reported a close correlation between autolysis and *pqsL* mutation, and found that a PAO1 Δ*pqsL* mutant overproduced PQS, leading to the conclusion that accumulation of this AQ is the primary cause of biofilm autolysis in the absence of functional PqsL (D’Argenio et al., 2002). This assumption has long been accepted since PQS is well known to play multiple roles in *P. aeruginosa* physiology. In fact, besides regulating its own production by controlling the expression of the AQ biosynthetic genes in complex with its cognate transcriptional regulator PqsR (Wade et al., 2005; Ilangovan et al., 2013), PQS also acts as an iron chelator and induces siderophore production (Bredenbruch et al., 2006; Diggle et al., 2007). Moreover, PQS contributes to the iron-starvation-independent and PqsR-independent signalling pathway regulating virulence gene expression (Rampioni et al., 2016). It also promotes the formation of membrane vesicles (MVs) in which PQS is both bioactive and bioavailable (Mashburn-Warren et al., 2008), and exhibits both pro- and anti-oxidant activities (Häussler and Becker, 2008).

Here we demonstrated for the first time that the lysis phenotype shown by the PAO1 Δ*pqsL* mutant is induced as a consequence of HHQ accumulation, that in turn leads to the transition of the Pf4 prophage to a lytic form *via* a PqsR-independent pathway. To the best of our knowledge, PqsR-independent activities have been described only for secreted HHQ, that displays antibacterial, antifungal, antimalarial and anticancer activities (Heeb et al., 2011; Saalim et al., 2020). Moreover, a series A AQ congener, likely different from HHQ and NHQ, modulates colony morphology in the clinical *P. aeruginosa* isolate ZK2870 also in the absence of PqsR (Gupta and Schuster, 2012). Interestingly, we observed that *P. aeruginosa* does not display the lysis phenotype when HHQ is produced at physiological levels, in accordance with the evidence that the primary role of HHQ is to activate the P*pqsA* promoter by interacting with PqsR (Rampioni et al., 2016). However, HHQ overproduction might generate stressful conditions causing cell lysis in mutant strains unable to synthesise HQNO.

Pf prophages, like many other phages, respond to oxidative stress. Indeed, exposure of *P. aeruginosa* to reactive oxygen species (ROS) and DNA damaging agents leads to the induction of prophages and to the formation of superinfective variants (Webb et al., 2003; Barraud et al., 2006; Hui et al., 2014). Furthermore, inhibition of cytochrome *bc*_1_ by HQNO also leads to ROS generation resulting in autolysis (Hazan et al., 2016). In *P. aeruginosa*, OxyR is the major oxidative stress regulator, controlling the expression of several genes including *katB*, *ahpB* and *ahpCF* (Wei et al., 2012). OxyR binds to the Pf4 genome (Wei et al., 2012; Secor et al., 2020) and may therefore be involved in phage induction during conditions of oxidative stress. However, we did not observe any variation in the transcriptional levels of the oxidative stress-regulated genes *katB*, *ahpB* and *ahpC* in colony biofilms of lytic mutants relative to non-lytic strains (**Figure S7**), strongly suggesting that HHQ overproduction does not induce Pf4 by causing oxidative stress. Interestingly, the SOS response-activating protein RecA is responsible for the conversion of the lambda phage from the lysogenic to the lytic cycle (Cox, 2007). Moreover, the SOS response induces error-prone DNA repair systems that could promote the emergence of superinfective Pf4 variants, that are often observed in *P. aeruginosa* biofilms (McElroy et al., 2014). Consequently, it could be hypothesised that HHQ accumulation drives transition of Pf4 to the lytic form by inducing stressful conditions that activate the SOS response. However, deletion of the *recA* gene, which is essential for the activation of the SOS response in *P. aeruginosa* (Mercolino et al., 2022), did not affect the autolysis induced by *pqsL* inactivation (**Figure S8**). This indicates that RecA and the SOS response are dispensable for this phenotype. The mechanism by which HHQ accumulation leads to the transition of Pf4 to a lytic form therefore remains unknown, and further studies will be needed. Although HQNO does not influence gene expression in *P. aeruginosa* during planktonic growth (Rampioni et al., 2016), as noted above, it does inhibit the cytochrome *bc*_1_ complex (Machan et al., 1992; Hazan et al., 2016). In the light of our observations, we propose that the production of HQNO may limit HHQ accumulation that otherwise would lead to colony biofilm autolysis.

While lysis is clearly detrimental to single bacterial cells, it provides a selective advantage to the bacterial population as a whole (Thomas and Hancock, 2009). As an example, a lysis phenotype could benefit in the context of a bacterial population organized in a biofilm community. Biofilm formation is considered an important survival strategy during the infection, since biofilms show increased tolerance to antibiotics and disinfectants, as well as resistance to phagocytosis and other components of the immune system, relative to planktonic cells (Høiby et al., 2010; McDougald et al., 2011; Flemming et al., 2016). Extracellular DNA (eDNA) is an essential constituent required for biofilm development (Whitchurch et al., 2002; Nemoto et al., 2003; Allesen-Holm et al., 2006; Yang et al., 2007), and plays an important role in the tolerance of *P. aeruginosa* biofilms toward positively charged antibiotics such as aminoglycosides and antimicrobial peptides (Mulcahy et al., 2008; Chiang et al., 2013). eDNA is released as a consequence of cell lysis and evidence has been provided that induction of lysis in *P. aeruginosa* involves the activation of autonomous selfish elements, such as prophages or endolysins (D’Argenio et al., 2002; Webb et al., 2003; Rice et al., 2009; Turnbull et al., 2016), as well as *via* HQNO-dependent ROS-induced membrane damage (Hazan et al., 2016).

Many different Pf prophages have been identified in *P. aeruginosa* (Hertveldt and Lavigne, 2008; Mai-Prochnow et al., 2015). Pf prophages are well-known to be associated with the formation of highly ordered liquid crystals promoting biofilm matrix organization in *P. aeruginosa* (Secor et al., 2015). In particular, Pf4 plays an essential role in the biofilm life cycle in *P. aeruginosa*. As *P. aeruginosa* biofilms develop, genes belonging to Pf4 prophage are among the most highly transcribed (Whiteley et al., 2001). In addition, Pf4 often mutates and converts to a superinfective variant able to re-infect and kill the *P. aeruginosa* parent strain, accompanied by the appearance of small colony variants in the dispersal population (Webb et al., 2003; Rice et al., 2009; Hui et al., 2014; McElroy et al., 2014). In this work, we demonstrated that Pf4 causes cell lysis in colony biofilms of PAO1 in response to HHQ accumulation. Similarly, reduced *pqsL* expression or PqsL functionality appear to be a common cause of autolysis in *P. aeruginosa* CF isolates. Nonetheless, further analyses will be needed to assess the involvement of Pf4 or other prophages in the lysis phenotype displayed by the CF strains, as Pf4 and many other Pf prophages, such as Pf1, Pf5 and Pf-LES, are frequently found among clinical isolates (Finnan et al., 2004; Kirov et al., 2007; Manos et al., 2008; Mathee et al., 2008; Winstanley et al., 2009; Fothergill et al., 2012; Knezevic et al., 2015; Ambroa et al., 2020).

It is noteworthy that besides exhibiting Pf4-mediated lysis in colony biofilms, the *P. aeruginosa* PAO1 Δ*pqsL* mutants are known to release more eDNA than the parental strain (Allesen-Holm et al., 2006; Yang et al., 2007). On this basis, it would be tempting to speculate that the *pqsL* mutation, by playing a role in the lysis phenotype of CF clinical isolates, confers the ability to form biofilms with greater tolerance to antibiotics *via* release of higher levels of eDNA. Future experiments are required to evaluate the possible impact of *pqsL* mutation and phage-mediated cell lysis on eDNA release and increased tolerance of *P. aeruginosa* biofilms to antibiotic treatment. The possible beneficial effect of having a mutated *pqsL* in the context of infection is consistent with recent studies reporting that the PqsL protein has the highest mutation rate among the QS genes analysed in a collection of clinical and environmental isolates (O’Connor et al., 2022). Taken together, our data highlight a close correlation between the *pqs* QS system, prophages and autolysis, that could possibly contribute to the formation of antibiotic resistant biofilms relevant in the context of CF chronic lung infections.

## Data availability statement

The original contributions presented in the study are included in the article/**Supplementary Material**. Further inquiries can be directed to the corresponding authors.

## Author contributions

GG, ML, MM, EF, NH, and GR performed the experiments and analysed the data. SH, MC, LL, PW, and GR contributed to conception and design of the study. GG and ML performed the statistical analysis. EF, SH, MC, PV, FI, LL, PW, and GR provided funding and resources. GG, ML, and GR wrote the first draft of the manuscript. FI and LL critically revised the first draft of the manuscript. All authors contributed to the article and approved the submitted version.
