# Supplementary material for: Alkyl-quinolone-dependent quorum sensing controls prophage-mediated autolysis in Pseudomonas aeruginosa colony biofilms
Source: Front Cell Infect Microbiol. 2023 May 26;13:1183681. doi: 10.3389/fcimb.2023.1183681 (PMC10250642; doi:10.3389/fcimb.2023.1183681)
Supplement: Supplementary file 1 [file DataSheet_1.pdf]

Alkyl-quinolone-dependent quorum sensing controls prophage-mediated autolysis in *Pseudomonas aeruginosa* colony biofilms

Giulia Giallonardi[1§†], Morgana Letizia[1†], Marta Mellini[1], Emanuela Frangipani[1#], Nigel Halliday[2], Stephan Heeb[2], Miguel Cámara[2], Paolo Visca[1,3,4], Francesco Imperi[1,3,4], Livia Leoni[1], Paul Williams[2*], Giordano Rampioni[1,3*]

[1] Department of Science, University Roma Tre, Rome, Italy; [2] National Biofilms Innovation Centre, Biodiscovery Institute and School of Life Sciences, University of Nottingham, Nottingham, United Kingdom; [3] IRCCS Fondazione Santa Lucia, Rome, Italy; [4] NBFC, National Biodiversity Future Center, Palermo, Italy.

Supplementary Material

Figure S1. Soft-agar lawns of *P. aeruginosa* PAO1-N wild type and mutant strains

Figure S2. Growth curves of *P. aeruginosa* PAO1-N wild type and mutant strains

Figure S3. Colony biofilms of *P. aeruginosa* mutant strains

Figure S4. P*pqsA* promoter activity in lytic and non-lytic strains

Figure S5. PCR analysis of *pqsL* in *P. aeruginosa* CF isolates

Figure S6. HHQ production in *P. aeruginosa* CF isolates

Figure S7. RT-qPCR analysis in lytic and non-lytic strains

Figure S8. Colony biofilms of *P. aeruginosa* mutant strains

Table S1. Bacterial strains used in this study

Table S2. Features of the CF clinical isolates analysed in this study

Table S3. Plasmids used in this study

Table S4. Oligonucleotides used in this study

References

Figure S1

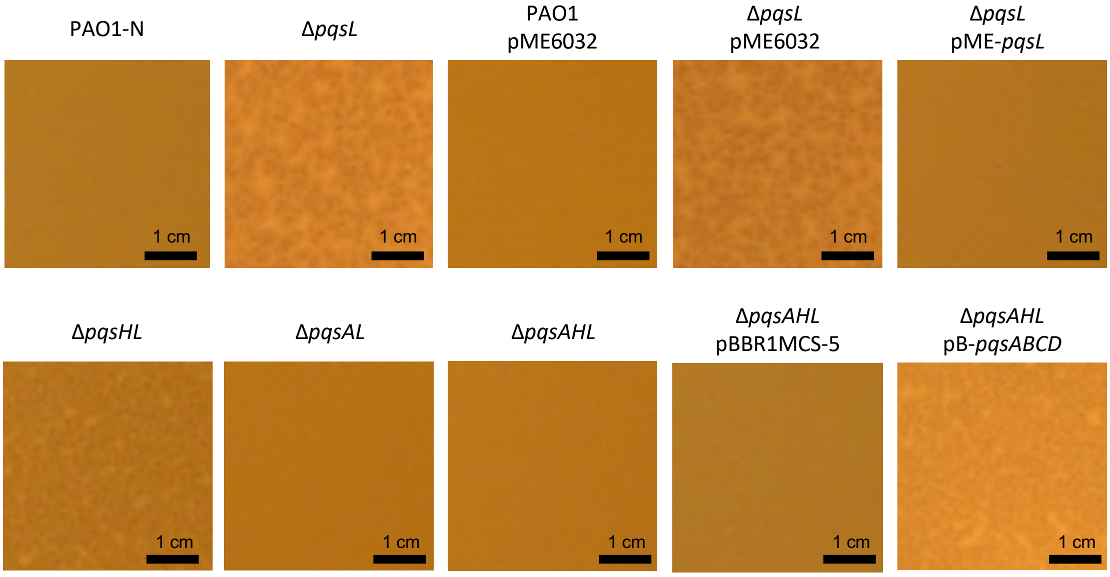

Figure S1. Soft-agar lawns of *P. aeruginosa* PAO1-N wild type and mutant strains

Enlarged section of Petri dishes containing soft-agar LB lawns formed by wild type *P. aeruginosa* PAO1-N or the indicated isogenic mutants. The medium was supplemented with 1 mM IPTG for the strains carrying the pME6032 or pME-*pqsL* plasmids. Representative pictures of three independent experiments are shown with scale bar (1 cm).

Figure S2

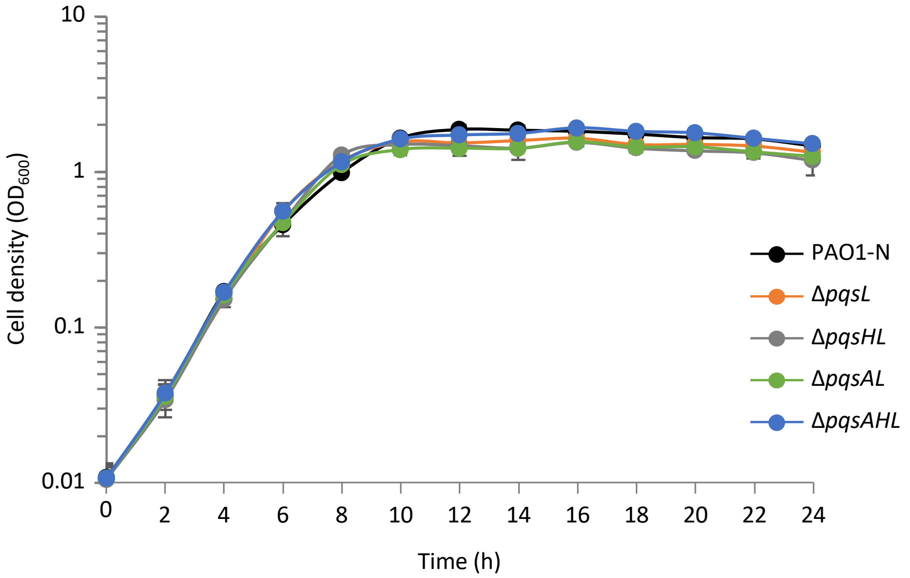

Figure S2. Growth curves of *P. aeruginosa* PAO1-N wild type and mutant strains

Growth curves of wild type *P. aeruginosa* PAO1-N or the indicated isogenic mutants grown in LB at 37°C with shaking (200 rpm). The average of three independent experiments is reported with SD.

Figure S3

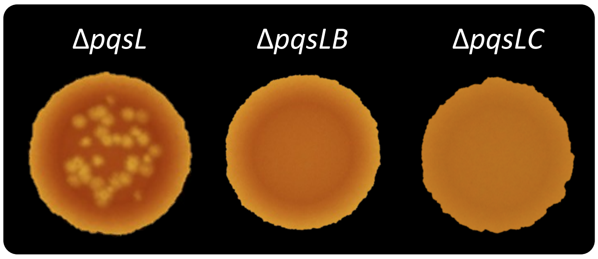

Figure S3. Colony biofilms of *P. aeruginosa* mutant strains

Colony biofilms on Congo-Red agar plates formed by the indicated *P. aeruginosa* PAO1-N mutants. Representative images of three independent experiments are shown.

Figure S4

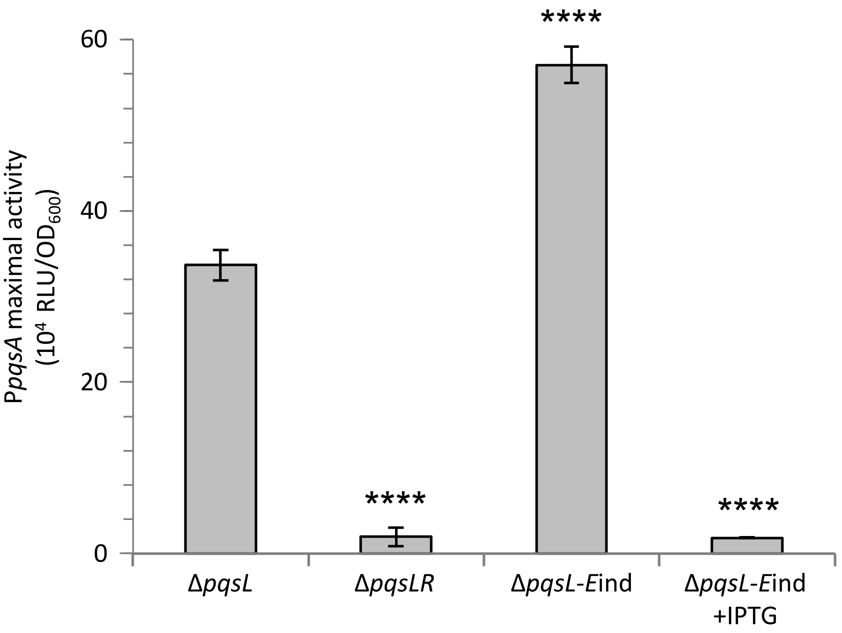

Figure S4. Effect of PqsR and PqsE on P*pqsA* promoter activity in the Δ*pqsL* mutant

Maximal promoter activity measured in the indicated *P. aeruginosa* PAO1-N mutant strains carrying the transcriptional fusions P*pqsA::lux*. Strains were grown in LB or in LB supplemented with 1 mM IPTG, as indicated below the histogram (+ IPTG). The average of three independent experiments is reported with standard deviation (SD). ****, $P < 0.0001$.

Figure S5

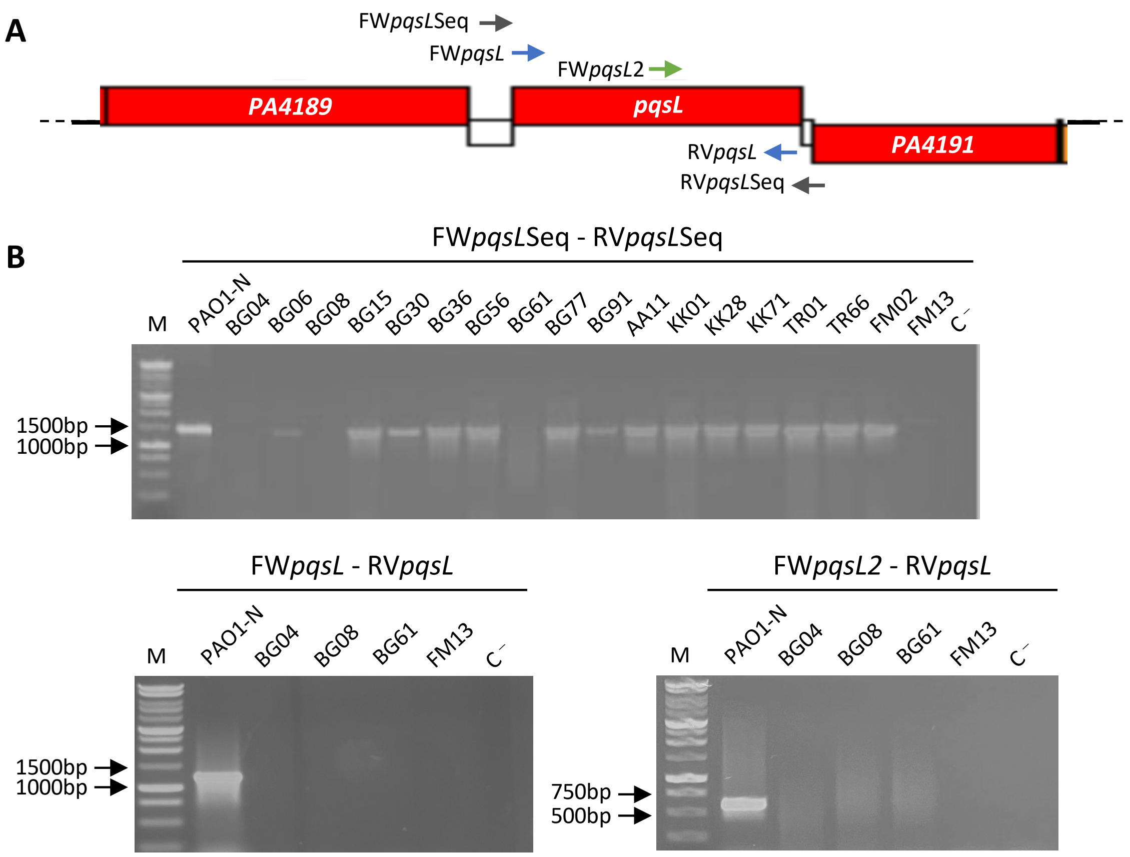

Figure S5. PCR analysis of *pqsL* in *P. aeruginosa* CF isolates

(A) Schematic representation of the *pqsL* gene *locus* in the genome of *P. aeruginosa* PAO1 and localization of the primer pairs used to PCR amplify *pqsL* in the *P. aeruginosa* PAO1-N strain and in CF lytic isolates. Image modified from the *Pseudomonas* Genome Database (Winsor *et al.*, 2011). (B) Gel electrophoresis analysis of PCR reactions performed with the indicated primer pairs using the genome of *P. aeruginosa* PAO1-N or of the indicated *P. aeruginosa* CF isolates as template. Expected amplicon sizes were: 1375 bp for the primer pair FW*pqsL*Seq - RV*pqsL*Seq; 1198 bp for the primer pair FW*pqsL* - RV*pqsL*; 572 bp for the primer pair FW*pqsL2* - RV*pqsL*. M, molecular weight marker Generuler 1 kb DNA ladder (GeneDirex); C-, no DNA template (negative control).

Figure S6

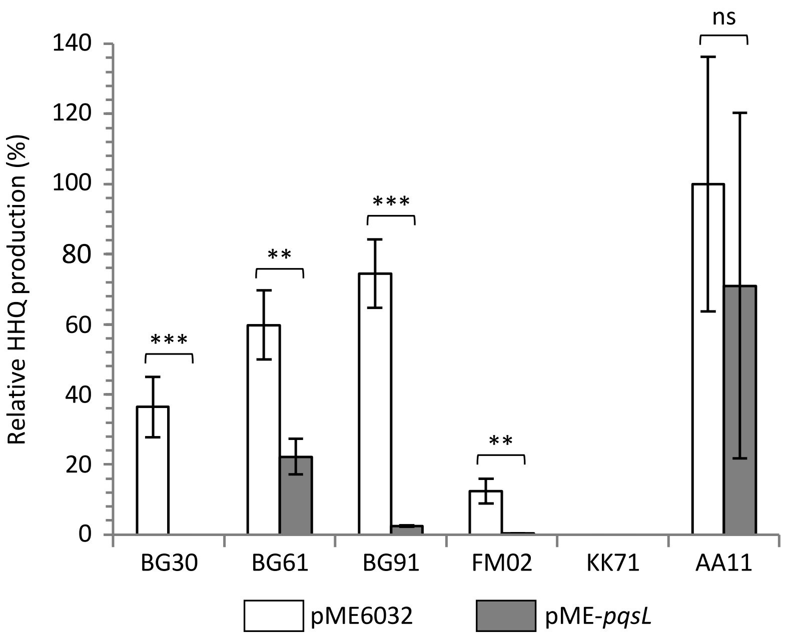

Figure S6. HHQ production in *P. aeruginosa* CF isolates

Histogram reporting the relative levels of HHQ measured by LC-MS/MS analysis in cell-free supernatants of the indicated CF isolates carrying the pME6032 empty plasmid (white bars) or the pME-*pqsL* plasmid for ectopic expression of *pqsL* (grey bars) grown in LB supplemented with 1 mM IPTG. The level of HHQ produced by the AA11(pME6032) strain was considered as 100%. The average of three independent experiments is reported with standard deviation (SD). **, P < 0.01; ***, P < 0.001; ns, not statistically significant. Levels of HHQ produced by the KK71(pME6032) and KK71(pME-*pqsL*) strains were below the detection limit.

Figure S7

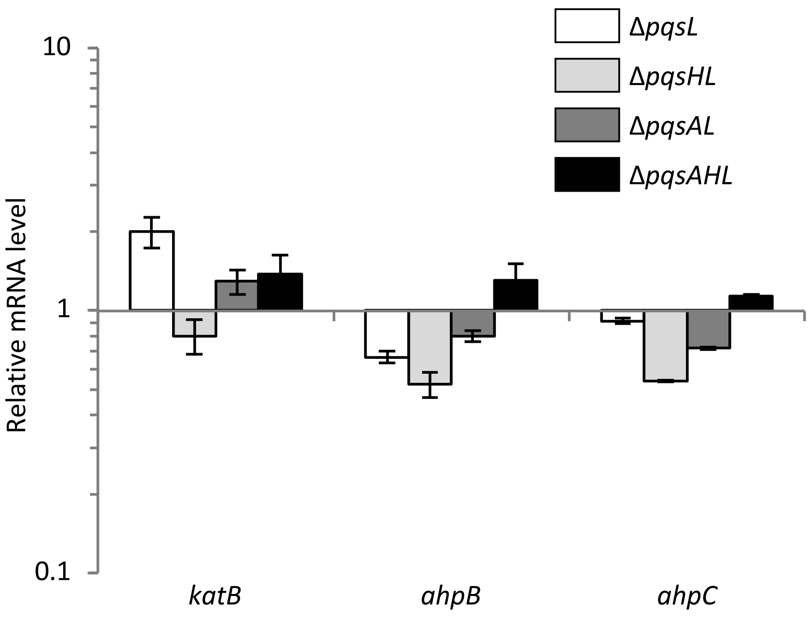

Figure S7. RT-qPCR analysis in lytic and non-lytic strains

Relative mRNA levels of the *katB*, *ahpB* and *ahpC* genes quantified by RT-qPCR in the *P. aeruginosa* Δ*pqsL* (white bars), Δ*pqsHL* (light grey bars), Δ*pqsAL* (dark grey bars), and Δ*pqsAHL* (black bars) mutant strains with respect to wild type PAO1-N. The average of three independent experiments is shown with standard deviations (SD).

Figure S8

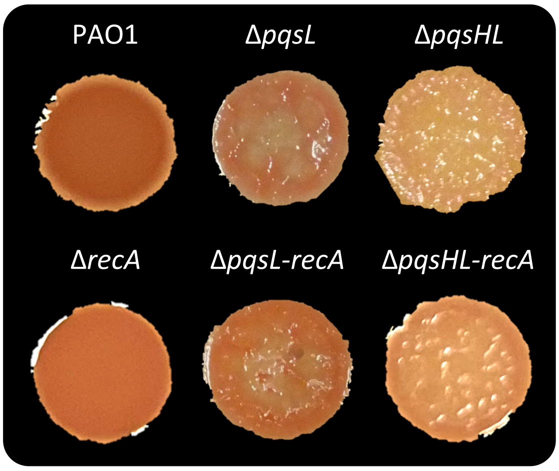

Figure S8. Colony biofilms of *P. aeruginosa* mutant strains

Colony biofilms on Congo-Red agar plates formed by the indicated *P. aeruginosa* PAO1-N mutants. Representative pictures of three independent experiments are shown.

Table S1. Bacterial strains used in this study

Strain	Relevant characteristics	Reference/Source
E. coli		
DH5α	Cloning strain.	Grant *et al.*, 1990
S17.1λ*pir*	Conjugative strain for suicide plasmids.	Simon *et al.*, 1983
P. aeruginosa		
PAO1-N	Nottingham collection wild type *P. aeruginosa* PAO1 strain.	
PAO1-N Δ*pqsA* *PpqsA::lux* AQ-Rep)	PAO1-N derivative strain deleted in the *pqsA* gene carrying chromosomal insertion of the *PpqsA::lux* transcriptional fusion; used for the detection of 2-alkyl-4-quinolones (AQs) signal molecules.	Fletcher *et al.*, 2007
Δ*pqsE*ind	PAO1-N derivative in which *pqsE* expression is under the control of a P*tac* promoter.	Rampioni *et al.*, 2010
Δ*pqsE*	PAO1-N strain carrying in frame clear deletion of the *pqsE* gene.	Rampioni *et al.*, 2010
Δ*pqsR*	PAO1-N strain carrying in frame clear deletion of the *pqsR* gene.	Rampioni *et al.*, 2016
PAO1-K	Wild type *P. aeruginosa* PAO1 strain, kindly provided by Prof. S. Kjelleberg (University of New South Wales, Australia).	Rice *et al.*, 2009
ΔPf4-K	*P. aeruginosa* PAO1-K mutant strain deleted in the Pf4 prophage genetic *locus*, kindly provided by Prof. S. Kjelleberg (University of New South Wales, Australia).	Rice *et al.*, 2009
Δ*pqsL*	PAO1-N strain carrying in frame clear deletion of the *pqsL* gene, obtained by allelic exchange using the plasmid pDM4Δ*pqsL*.	This study
Δ*pqsHL*	Δ*pqsL* strain carrying in frame clear deletion of the *pqsH* gene, obtained by allelic exchange using the plasmid pDM4Δ*pqsH*.	This study
Δ*pqsAL*	Δ*pqsL* strain carrying in frame clear deletion of the *pqsA* gene, obtained by allelic exchange using the plasmid pDM4Δ*pqsA*.	This study
Δ*pqsAHL*	Δ*pqsHL* strain carrying in frame clear deletion of the *pqsA* gene, obtained by allelic exchange using the plasmid pDM4Δ*pqsA*.	This study
Δ*pqsLB*	Δ*pqsL* strain carrying in frame clear deletion of the *pqsB* gene, obtained by allelic exchange using the plasmid pDM4Δ*pqsB*.	This study
Δ*pqsLC*	Δ*pqsL* strain carrying in frame clear deletion of the *pqsC* gene, obtained by allelic exchange using the plasmid pDM4Δ*pqsC*.	This study
Δ*pqsLR*	Δ*pqsL* strain carrying in frame clear deletion of the *pqsR* gene, obtained by allelic exchange using the plasmid pDM4Δ*pqsR*.	This study
Δ*pqsHLR*	Δ*pqsHL* strain carrying in frame clear deletion of the *pqsR* gene, obtained by allelic exchange using the plasmid pDM4Δ*pqsR*.	This study
Δ*pqsL-E*ind	Δ*pqsE*ind strain carrying in frame clear deletion of the *pqsL* gene, obtained by allelic exchange using the plasmid pDM4Δ*pqsL*.	This study
Δ*pqsHL-E*ind	Δ*pqsL-E*ind strain carrying in frame clear deletion of the *pqsH* gene, obtained by allelic exchange using the plasmid pDM4Δ*pqsH*.	This study
Δ*pqsL*-K	PAO1-K strain carrying in frame clear deletion of the *pqsL* gene, obtained by allelic exchange using the plasmid pDM4Δ*pqsL*.	This study
Δ*pqsL*-Pf4-K	ΔPf4-K strain carrying in frame clear deletion of the *pqsL* gene, obtained by allelic exchange using the plasmid pDM4Δ*pqsL*.	This study
Δ*recA*	PAO1-N strain carrying in frame clear deletion of the *recA* gene, obtained by allelic exchange using the plasmid pDM4Δ*recA*.	This study
Δ*pqsL-recA*	Δ*pqsL* strain carrying in frame clear deletion of the *recA* gene, obtained by allelic exchange using the plasmid pDM4Δ*recA*.	This study
Δ*pqsHL-recA*	Δ*pqsHL* strain carrying in frame clear deletion of the *recA* gene, obtained by allelic exchange using the plasmid pDM4Δ*recA*.	This study

Table S2. Features of the CF clinical isolates analysed in this study

Isolate	Lysis phenotype	*pqsL* amplification	PqsL amino acid substitution(s)	Reference/Source
BG02	–	nd	nd	Imperi *et al.*, 2019
BG04	+	–	nd	Imperi *et al.*, 2019
BG06	+	+	E330G	Imperi *et al.*, 2019
BG08	+	–	nd	Imperi *et al.*, 2019
BG11	–	nd	nd	Imperi *et al.*, 2019
BG15	+	+	E330G	Imperi *et al.*, 2019
BG18	–	nd	nd	Imperi *et al.*, 2019
BG21	–	nd	nd	Imperi *et al.*, 2019
BG22	–	nd	nd	Imperi *et al.*, 2019
BG24	–	nd	nd	Imperi *et al.*, 2019
BG25	–	nd	nd	Imperi *et al.*, 2019
**BG30**	+	+	D175E R179H S206A A232S	Imperi *et al.*, 2019
BG33	–	nd	nd	Imperi *et al.*, 2019
BG36	+	+	-	Imperi *et al.*, 2019
BG41	–	nd	nd	Imperi *et al.*, 2019
BG50	–	nd	nd	Imperi *et al.*, 2019
BG51	–	nd	nd	Imperi *et al.*, 2019
BG56	+	+	-	Imperi *et al.*, 2019
**BG61**	+	–	nd	Imperi *et al.*, 2019
BG64	–	nd	nd	Imperi *et al.*, 2019
BG68	–	nd	nd	Imperi *et al.*, 2019
BG75	–	nd	nd	Imperi *et al.*, 2019
BG76	–	nd	nd	Imperi *et al.*, 2019
BG77	+	+	-	Imperi *et al.*, 2019
BG87	–	nd	nd	Imperi *et al.*, 2019
BG89	–	nd	nd	Imperi *et al.*, 2019
BG90	–	nd	nd	Imperi *et al.*, 2019
**BG91**	+	+	D175E E330G	Imperi *et al.*, 2019
BG93	–	nd	nd	Imperi *et al.*, 2019
BG94	–	nd	nd	Imperi *et al.*, 2019
BG97	–	nd	nd	Imperi *et al.*, 2019
BG98	–	nd	nd	Imperi *et al.*, 2019
AA02	–	nd	nd	Bragonzi *et al.*, 2009
**AA11**	+	+	D117E D175E R179H A232S	Bragonzi *et al.*, 2009
AA12	–	nd	nd	Bragonzi *et al.*, 2009
BT02	–	nd	nd	Bragonzi *et al.*, 2009
BT73	–	nd	nd	Bragonzi *et al.*, 2009
KK01	+	+	E330G	Bragonzi *et al.*, 2009
KK02	–	nd	nd	Bragonzi *et al.*, 2009
KK27	–	nd	nd	Bragonzi *et al.*, 2009
KK28	+	+	E330G	Bragonzi *et al.*, 2009
**KK71**	+	+	E330G	Bragonzi *et al.*, 2009
TR01	+	+	E330G	Bragonzi *et al.*, 2009
TR66	+	+	E330G	Bragonzi *et al.*, 2009

Isolate	Lysis phenotype	*pqsL* amplification	PqsL amino acid substitution(s)	Reference/Source
**FM02**	+	+	-	Massai *et al.*, 2011
FM11	–	nd	nd	Massai *et al.*, 2011
FM12	–	nd	nd	Massai *et al.*, 2011
FM13	+	–	nd	Massai *et al.*, 2011
FM14	–	nd	nd	Massai *et al.*, 2011
FM15	–	nd	nd	Massai *et al.*, 2011

nd, not determined. The 6 representative lytic strains complemented by ectopic expression of *pqsL via* pME-*pqsL* are in bold.

Table S3. Plasmids used in this study

Plasmid	Relevant characteristics and plasmid contruction	Reference/Source
pDM4	Suicide vector; *sacBR*; *oriR6K*; Cm^R.	Milton *et al.*, 1996
pDM4Δ*pqsL*	pDM4 derivative plasmid for *pqsL* in-frame deletion; Cm^R.	Rampioni *et al.*, 2016
pDM4Δ*pqsH*	pDM4 derivative plasmid for *pqsH* in-frame deletion; Cm^R.	Fletcher *et al.*, 2007
pDM4Δ*pqsA*	pDM4 derivative plasmid for *pqsA* in-frame deletion; Cm^R.	Aendekerk *et al.*, 2005
pDM4Δ*pqsR*	pDM4 derivative plasmid for *pqsR* in-frame deletion; Cm^R.	Ilangovan *et al.*, 2013
pDM4Δ*recA*	pDM4 derivative plasmid for *recA* in-frame deletion; Cm^R.	Scala *et al.*, 2020
pBBR1MCS-5	Vector for constitutive gene expression; Gm^R.	Kovach *et al.*, 1995
pBBR-*pqsABCD*	Plasmid derived from pBBR1MCS-2 for constitutive expression of the *pqsABCD* operon; Km^R. Kindly provided by Prof. S. Fetzner (University of Munster, Germany).	Niewerth *et al.*, 2011
pME6032	Vector for IPTG-inducible gene expression; *lacI*^{Q}, Tc^R.	Heeb *et al.*, 2002
miniCTX-P*pqsA::lux*	miniCTX-lux derivative used to insert the P*pqsA::lux* fusion in the chromosome of different *P. aeruginosa* strains; Tc^R.	Diggle *et al.*, 2007
pDM4Δ*pqsB*	pDM4-derived plasmid for the generation of the PAO1 Δ*pqsLB* mutant strain. It contains the DNA fragments encompassing the upstream region of *pqsB* gene originated with primers FW*pqsB*UP and RV*pqsB*UP (**Table S4**) and the downstream region of the *pqsB* gene originated with primers FW*pqsB*DW and RV*pqsB*DW (**Table S4**), and cloned in pDM4 by XhoI-XbaI.	This study
pDM4Δ*pqsC*	pDM4-derived plasmid for the generation of the PAO1 Δ*pqsLC* mutant strain. It contains the DNA fragments encompassing the upstream region of *pqsB* gene originated with primers FW*pqsC*UP and RV*pqsC*UP (**Table S4**) and the downstream region of the *pqsB* gene originated with primers FW*pqsC*DW and RV*pqsC*DW (**Table S4**), and cloned in pDM4 by XhoI-XbaI.	This study
pB-*pqsABCD*	pBBR1MCS-5 derived plasmid for constitutive expression of the *pqsABCD* operon. Obtained by subcloning the DNA region encompassing the *pqsABCD* operon from pBBR-*pqsABCD* in pBBR1MCS-5 by using the restriction enzymes SalI-SacI.	This study
pME-*pqsL*	pME6032-derivative plasmid for IPTG-inducible expression of *pqsL.* Obtained by cloning in pME6032 with the restriction enzymes EcoRI-SalI a DNA region encompassing the *pqsL* gene amplified with primers FW*pqsL* and RV*pqsL* (**Table S4**).	This study

Table S4. Oligonucleotides used in this study

Name	Sequence (5'-3')[a]	Restriction site
FW*pqsB*UP	CCGCTCGAGCGAAGGACACACTATCGAG	XhoI
RV*pqsB*UP	CCGGAATTCAATCAACATGCCCGTTCCTC	EcoRI
FW*pqsB*DW	CCGGAATTCCATGCATAAGGTCAAACTGGC	EcoRI
RV*pqsB*DW	TGCTCTAGACGCGAGGTGAAGTCGAGC	XbaI
FW*pqsC*UP	CCGCTCGAGCGACCAGGGCTATCGCA	XhoI
RV*pqsC*UP	CCGGAATTCCTTATGCATGAGCTTCTCC	EcoRI
FW*pqsC*DW	CCGGAATTCTGCTGAGGCATCGCCATGT	EcoRI
RV*pqsC*DW	TGCTCTAGACCGAGCAGGATCGACAGG	XbaI
FW*pqsL*	CCGGAATTCATGACGGACAACCATATCG	EcoRI
RV*pqsL*	TACGAGCTCGCTGGCGGGTTCAGCCG	SalI
FW*pqsL*Seq	CAAGTGCAGACAACACCCATT	–
RV*pqsL*Seq	GTGGCCAAGGTATTCCCGC	–
FW*pqsL2*	GGCTGGCCTACTTCTATCCG	–
FW16SRT	GAGAGTTTGATCCTGGCTCAG	–
RV16SRT	CTACGGCTACCTTGTTACGA	–
FW*katB*RT	TGTATTCCAACGAGGGCACC	–
RV*katB*RT	GCTCACCAGGTCATTGGTCA	–
FW*ahpB*RT	GACTCGCAGTTCACCCATCA	–
RV*ahpB*RT	TTCACCACTTGGTGCTGGAC	–
FW*ahpC*RT	AACGGCAAGTTCATCGAGGT	–
RV*ahpC*RT	AGACCTTGTGCGAGAAGTGG	–

[a] Restriction sites are underlined in the oligonucleotide sequences.

References

- Aendekerk, S., Diggle, S.P., Song, Z., Høiby, N., Cornelis, P., Williams, P., et al. (2005). The MexGHI-OpmD multidrug efflux pump controls growth, antibiotic susceptibility and virulence in *Pseudomonas aeruginosa via* 4-quinolone-dependent cell-to-cell communication. *Microbiology.* 151:1113-1125. doi: 10.1099/mic.0.27631-0
- Bragonzi, A., Paroni, M., Nonis, A., Cramer, N., Montanari, S., Rejman, J., et al. (2009). *Pseudomonas aeruginosa* microevolution during cystic fibrosis lung infection establishes clones with adapted virulence. *Am. J. Respir. Crit. Care. Med.* 180:138-145. doi: 10.1164/rccm.200812-1943OC
- Diggle, S.P., Matthijs, S., Wright, V.J., Fletcher, M.P., Chhabra, S.R., Lamont, I.L., et al. (2007). The *Pseudomonas aeruginosa* 4-quinolone signal molecules HHQ and PQS play multi- functional roles in quorum sensing and iron entrapment. *Chem. Biol.* 14: 87-96. doi: 10.1016/j.chembiol.2006.11.014
- Fletcher, M.P., Diggle, S.P., Crusz, S.A., Chhabra, S.R., Cámara, M., Williams, P. (2007). A dual biosensor for 2-alkyl-4-quinolone quorum-sensing signal molecules. *Environ. Microbiol.* 9:2683-2693. doi: 10.1111/j.1462-2920.2007.01380.x
- Grant, S.G., Jessee, J., Bloom, F.R., Hanahan, D. (1990). Differential plasmid rescue from transgenic mouse DNAs into *Escherichia coli* methylation-restriction mutants. *Proc. Natl. Acad. Sci. USA.* 87:4645-4649. doi: 10.1073/pnas.87.12.4645
- Heeb, S., Blumer, C., Haas, D. (2002). Regulatory RNA as mediator in GacA/RsmA-dependent global control of exoproduct formation in *Pseudomonas fluorescens* CHA0. *J. Bacteriol.* 184:1046-1056. doi: 10.1128/jb.184.4.1046-1056.2002
- Ilangovan, A., Fletcher, M., Rampioni, G., Pustelny, C., Rumbaugh, K., Heeb, S., et al. (2013). Structural basis for native agonist and synthetic inhibitor recognition by the *Pseudomonas aeruginosa* quorum sensing regulator PqsR (MvfR). *PLoS. Pathog*. 9:e1003508. doi: 10.1371/journal.ppat.1003508
- Imperi, F., Fiscarelli, E.V., Visaggio, D., Leoni, L., Visca, P. (2019). Activity and impact on resistance development of two antivirulence fluoropyrimidine drugs in *Pseudomonas aeruginosa*. *Front. Cell. Infect. Microbiol.* 9:49. doi: 10.3389/fcimb.2019.00049
- Kovach, M.E., Elzer, P.H., Hill, D.S., Robertson, G.T., Farris, M.A., Roop, R.M. 2nd., et al. (1995). Four new derivatives of the broad-host-range cloning vector pBBR1MCS, carrying different antibiotics-resistance cassettes. *Gene.* 166:175-176. doi: 10.1016/0378-1119(95)00584-1

- Massai, F., Imperi, F., Quattrucci, S., Zennaro, E., Visca, P., Leoni, L. (2011). A multitask biosensor for micro-volumetric detection of *N*-3-oxo-dodecanoyl-homoserine lactone quorum sensing signal. *Biosens. Bioelectron.* 26:3444-3449. doi: 10.1016/j.bios.2011.01.022
- Milton, D.L., O'Toole, R., Horstedt, P., Wolf-Watz, H. (1996). Flagellin A is essential for the virulence of *Vibrio anguillarum*. *J. Bacteriol.* 178:1310-1319. doi: 10.1128/jb.178.5.1310-1319.1996
- Niewerth, H., Bergander, K., Chhabra, S.R., Williams, P., Fetzner, S. (2011). Synthesis and biotransformation of 2-alkyl-4*(1H)*-quinolones by recombinant *Pseudomonas putida* KT2440. *Appl. Microbiol. Biotechnol.* 91:1399-1408. doi: 10.1007/s00253-011-3378-0
- Rampioni, G., Falcone, M., Heeb, S., Frangipani, E., Fletcher, M.P., Dubern, J.F., et al. (2016). Unravelling the genome-wide contributions of specific 2-alkyl-4-quinolones and PqsE to quorum sensing in *Pseudomonas aeruginosa*. *PLoS. Pathog.* 12:e1006029. doi: 10.1371/journal.ppat.1006029
- Rampioni, G., Pustelny, C., Fletcher, M.P., Wright, V.J., Bruce, M., Rumbaugh, K.P., et al. (2010). Transcriptomic analysis reveals a global alkyl-quinolone-independent regulatory role for PqsE in facilitating the environmental adaptation of *Pseudomonas aeruginosa* to plant and animal hosts. *Environ. Microbiol.* 12:1659-1673. doi: 10.1111/j.1462-2920.2010.02214.x
- Rice, S.A., Tan, C.H., Mikkelsen, P.J., Kung, V., Woo, J., Tay, M., et al. (2009). The biofilm life cycle and virulence of *Pseudomonas aeruginosa* are dependent on a filamentous prophage. *ISME. J.* 3:271-282. doi: 10.1038/ismej.2008.109
- Scala, R., Di Matteo, A., Coluccia, A., Lo Sciuto, A., Federici, L., Travaglini-Allocatelli, C., et al. (2020). Mutational analysis of the essential lipopolysaccharide-transport protein LptH of *Pseudomonas aeruginosa* to uncover critical oligomerization sites. *Sci. Rep.* 10:11276. doi: 10.1038/s41598-020-68054-7
- Simon, R., Priefer, U., Puhler, A. (1983). A broad host range mobilization system for *in vivo* genetic-engineering: transposon mutagenesis in Gram-negative bacteria. *Biotechnology.* 1:784-791. doi: 10.1038/nbt1183-784
- Winsor, G.L., Lam, D.K., Fleming, L., Lo, R., Whiteside, M.D., Yu, N.Y., et al. (2011). *Pseudomonas* Genome Database: improved comparative analysis and population genomics capability for *Pseudomonas* genomes. *Nucleic. Acids. Res.* 39:D596-600. doi: 10.1093/nar/gkq869
